# Burst Firing Enhances Neural Output Correlation

**DOI:** 10.3389/fncom.2016.00042

**Published:** 2016-05-09

**Authors:** Ho Ka Chan, Dong-Ping Yang, Changsong Zhou, Thomas Nowotny

**Affiliations:** ^1^Centre for Computational Neuroscience and Robotics, School of Engineering and Informatics, University of SussexBrighton, UK; ^2^Department of Physics, Hong Kong Baptist UniversityKowloon Tong, Hong Kong; ^3^Centre for Nonlinear Studies, Institute of Computational and Theoretical Studies, Hong Kong Baptist UniversityKowloon Tong, Hong Kong; ^4^School of Physics, University of SydneyNew South Wales, Sydney, NSW, Australia

**Keywords:** burst, correlation, synaptic filtering, leaky integrate-and-fire, adaptation

## Abstract

Neurons communicate and transmit information predominantly through spikes. Given that experimentally observed neural spike trains in a variety of brain areas can be highly correlated, it is important to investigate how neurons process correlated inputs. Most previous work in this area studied the problem of correlation transfer analytically by making significant simplifications on neural dynamics. Temporal correlation between inputs that arises from synaptic filtering, for instance, is often ignored when assuming that an input spike can at most generate one output spike. Through numerical simulations of a pair of leaky integrate-and-fire (LIF) neurons receiving correlated inputs, we demonstrate that neurons in the presence of synaptic filtering by slow synapses exhibit strong output correlations. We then show that burst firing plays a central role in enhancing output correlations, which can explain the above-mentioned observation because synaptic filtering induces bursting. The observed changes of correlations are mostly on a long time scale. Our results suggest that other features affecting the prevalence of neural burst firing in biological neurons, e.g., adaptive spiking mechanisms, may play an important role in modulating the overall level of correlations in neural networks.

## Introduction

Many *in vivo* studies have revealed that neurons in a variety of brain areas frequently exhibit correlated activity (Zohary et al., 1994; König and Engel, 1995; Bair et al., 2001; Kohn and Smith, 2005; Okun and Lampl, 2008; Gerkin et al., 2013). However, the functions and consequences of correlations, and whether correlated input may carry any information have long been debated (Shadlen and Newsome, 1998; Panzeri et al., 1999; Salinas et al., 2001; Averbeck et al., 2006; Wolfe et al., 2010; Dipoppa and Gutkin, 2013). One of the key questions is how input correlations are processed and transmitted from a layer of neurons to the next (Shadlen and Newsome, 1998; Diesmann et al., 1999; Salinas and Sejnowski, 2000; Reyes, 2003; de la Rocha et al., 2007; Ostojic et al., 2009; Litwin-Kumar et al., 2011; Hong et al., 2012; Schultze-Kraft et al., 2013).

The conductance based LIF model (Stein, 1967) is often used in numerical and analytical studies of neural dynamics. However, it is very challenging to analytically derive the higher order statistics of output spike trains in this model, due to nonlinearity in the model and the resulting neural computations. Most previous work has studied the problem of correlation transfer by resorting to further approximations of single neuron dynamics and considering the pairwise correlation between two neurons receiving correlated inputs. A typical strategy is to use the diffusion approximation, mimicking the synaptic inputs by currents with Gaussian white noise (de la Rocha et al., 2007; Ostojic et al., 2009; Litwin-Kumar et al., 2011; Hong et al., 2012; Schultze-Kraft et al., 2013). This approach assumes that autocorrelations in the inputs are small. However, biological neurons may have slow synapses, in which ion channels take a substantial time to close after opening. This results in synaptic filtering and introduces autocorrelations in the inputs, rendering the assumption of small autocorrelations invalid.

In order to understand how input features, e.g., the level of background activity or input synchrony, affect correlation transfer, Ostojic et al. (2009) and Schultze-Kraft et al. (2013) studied changes of the membrane potential distribution of a neuron, and hence its probability of firing, in response to an additional input spike; Rosenbaum and Josić (2011) studied the conditional probability of a neuron to fire given that the other neuron has just recently fired. These approaches make the assumption that an input spike can contribute to at most one output spike, which is also problematic when neurons with slow synapses are considered, as synaptic filtering by slow synapses may induce burst firing (Moreno-Bote and Parga, 2004). The effects of synaptic filtering, and in particular the resulting burst firing, on neural correlation transfer is little known.

In this work, we aimed to study the role of synaptic filtering in correlation transfer by numerical simulations of a pair of LIF neurons receiving partially overlapping inputs. We found that neurons with slow synapses exhibit unexpectedly strong output correlations of a long time scale and at the same time fire in a strong bursting pattern. When controlling the amount of burst firing by incorporating biologically realistic spike adaptation mechanisms, namely after-spike hyperpolarizing currents (AHP) (Storm, 1987, 1990) and/or variable firing thresholds (Henze and Buzsáki, 2001; Platkiewicz and Brette, 2010), we observed that burst firing greatly enhances output correlations of a long time scale. But it only modestly increases output correlations at a shorter time scale, which correspond to synchrony. In the remainder of the work, we will refer to correlations at long time scales as “correlations” and correlations at short time scales simply as “synchrony,” unless otherwise specified. Furthermore, the “slowness” of synapses is always understood to be in comparison to the time scale of membrane potential integration.

## Methods

### Neuron model

Neural dynamics are simulated using the conductance-based LIF model (Stein, 1967). The dynamics of membrane potential are given by:

(1) $$\begin{array}{rcl} C\frac{d}{dt}V\left(t\right) & + & \left[V\left(t\right)-V_{e}\right]G_{e}\left(t\right)+\left[V\left(t\right)-V_{i}\right]G_{i}\left(t\right)\\ +\left[V\left(t\right)-V_{l}\right]G_{l}+I_{fahp}+I_{sahp}=0, \end{array}$$

where *C* is the membrane capacitance, *V*(*t*) is the membrane potential, *V*_*l*_, *V*_*e*_ and *V*_*i*_ are the membrane resting potential, reversal potential of excitatory synapses and that of inhibitory synapses, respectively. *G*_*l*_ is the membrane leak conductance. *I*_*fahp*_ and *I*_*sahp*_ are fast and slow after-spike hyperpolarizing currents (See Section Membrane Potential Reset and After-Spike Hyperpolarization). When the membrane potential reaches the firing threshold *V*_*th*_, the neuron fires a spike, and then the membrane potential is reset to the potential *V*_*reset*_ and clamped to that value for a fixed refractory period *t*_*refract*_.

Inputs are modeled by conductances. The excitatory and inhibitory synaptic conductances, denoted by *G*_*e*_(*t*) and *G*_*i*_(*t*) respectively, are modeled by linear summation of conductance changes induced by each presynaptic incoming spike.

To facilitate the interpretation of the neuron dynamics, we can separate the synaptic conductance into tonic parts and fluctuating parts (Richardson and Gerstner, 2005) and rewrite equation (1) (excluding the AHP currents) to

(2) $$\begin{array}{rcl} \tau_{eff}\frac{d}{dt}V\left(t\right) & = & -\left(V\left(t\right)-V_{0}\right)-\frac{G_{e_{f}}\left(t\right)}{G_{total}}\left[V\left(t\right)-V_{e}\right]\\ -\frac{G_{i_{f}}\left(t\right)}{G_{total}}\left[V\left(t\right)-V_{i}\right], \end{array}$$

where *G*_*total*_ = *G*_*l*_ + 〈*G*_*e*_(*t*)〉+〈*G*_*i*_(*t*)〉 with 〈·〉 denoting the average over a long period of time, τ_*eff*_ = *C*∕*G*_*total*_, *V*_0_ = [*V*_*l*_*G*_*l*_ + *V*_*e*_ 〈 *G*_*e*_(*t*)〉+*V*_*i*_ 〈 *G*_*i*_(*t*)〉]∕*G*_*total*_ and *G*_*s*_*f*__(*t*) = *G*_*s*_(*t*) − 〈*G*_*s*_(*t*)〉, where the subscript *s* can be chosen as *e*, referring to “excitatory” or *i*, referring to “inhibitory.”

τ_*eff*_ in Equation (2) denotes the effective membrane time constant, which quantifies how fast the membrane responds to the fluctuating conductances and is related to the total synaptic conductance which depends on the level of average input activities. *V*_0_ refers to the mean membrane potential when spiking dynamics are ignored (Kuhn et al., 2004). The simulations reported below were run using equation (1), but we will refer to equation (2) and in particular the value of τ_*eff*_ in the interpretation of the results.

### Spiking mechanism

In this work, the prevalence of neural burst firing is varied using different spiking mechanisms.

#### Firing threshold

In Sections Asymmetric Effects of Membrane Potential and Synaptic Integration Time Constants on Output Correlations and Slow Synaptic Filtering Induces Strong Burst Firing, a hard firing threshold is applied, which means the firing threshold is fixed at a constant value *V*_*th*_*rest*__.

In Section Burst Firing Greatly Enhances Output Correlations, a soft firing threshold is applied. The firing threshold is raised to *V*_*th*_*max*__ right after firing or at the end of the hard refractory period, if it is incorporated. The firing threshold *V*_*th*_ then decays exponentially to a rest value *V*_*th*_*rest*__ as described in Clopath et al. (2010):

(3) $$\begin{array}{l} \tau_{th}\frac{d}{dt}V_{th}=-\left(V_{th}-V_{{th}_{rest}}\right), \end{array}$$

where τ_*th*_ is the threshold decay time constant.

When the soft firing threshold is applied, neurons need to depolarize more in order to fire successive spikes within a short period. Thus, it can be expected that the adaptive threshold can suppress burst firing that otherwise would occur.

#### Membrane potential reset and after-spike hyperpolarization

In Sections Asymmetric Effects of Membrane Potential and Synaptic Integration Time Constants on Output Correlations and Slow Synaptic Filtering Induces Strong Burst Firing, the membrane potential is brought to a reset potential *V*_*reset*_ right after firing and clamped to that value for a fixed refractory period *t*_*refract*_. In such cases *I*_*fahp*_ = *I*_*sahp*_ = 0 at all time.

In Section Burst Firing Greatly Enhances Output Correlations, a more realistic reset mechanism is incorporated. The membrane potential is raised to *V*_*spike*_ right after crossing the firing threshold, mimicking the run-away rise of membrane potential in Hodgkin-Huxley neurons during a spike. Then, after a time delay *t*_*delay*_, *I*_*fahp*_ and *I*_*sahp*_ are set to *I*_*fahp*_*max*__ and *I*_*sahp*_*max*__ respectively, mimicking the onset of two different after-spike hyperpolarization or repolarization currents (AHP). They then decay exponentially as described in Clopath et al. (2010)

(4) $$\begin{array}{l} \tau_{xahp}\frac{d}{dt}I_{xahp}=-I_{xahp}, \end{array}$$

where the subscript *x* can be *f*, referring to “fast” or *s*, referring to “slow.” τ_*fahp*_ (τ_*sahp*_) (refers to the fast (slow) AHP decay time constant. The AHP currents are applied in addition to the soft firing threshold described above.

### Synaptic input

The contribution of each input to the conductance change is modeled by an alpha function and the integration (from *t* = −∞ to ∞) of conductance change due to an input spike is kept constant by multiplication of an additional factor of $\frac{1}{\tau_{s}}$. The total conductance change is modeled by linear summation of conductance change due to each presynaptic input spike.

(5) $$\begin{array}{l} g_{s}\left(t\right)=A_{s}\frac{t}{{\tau_{s}}^{2}}e^{1-\frac{t}{\tau_{s}}}H\left(t\right),\;G_{s}\left(t\right)=\sum_{j}g_{s}\left(t-t_{j}\right), \end{array}$$

where *A*_*s*_ are synaptic efficacies, τ_*s*_ are synaptic time constant and the subscript s can be chosen as e, referring to “excitatory” or *i*, referring to “inhibitory.” *H*(*t*) is the Heaviside step function. The times *t*_*j*_ are referring to the timing of input spikes, which is assumed to have Poisson statistics.

### Input correlation

**Figure 1:**
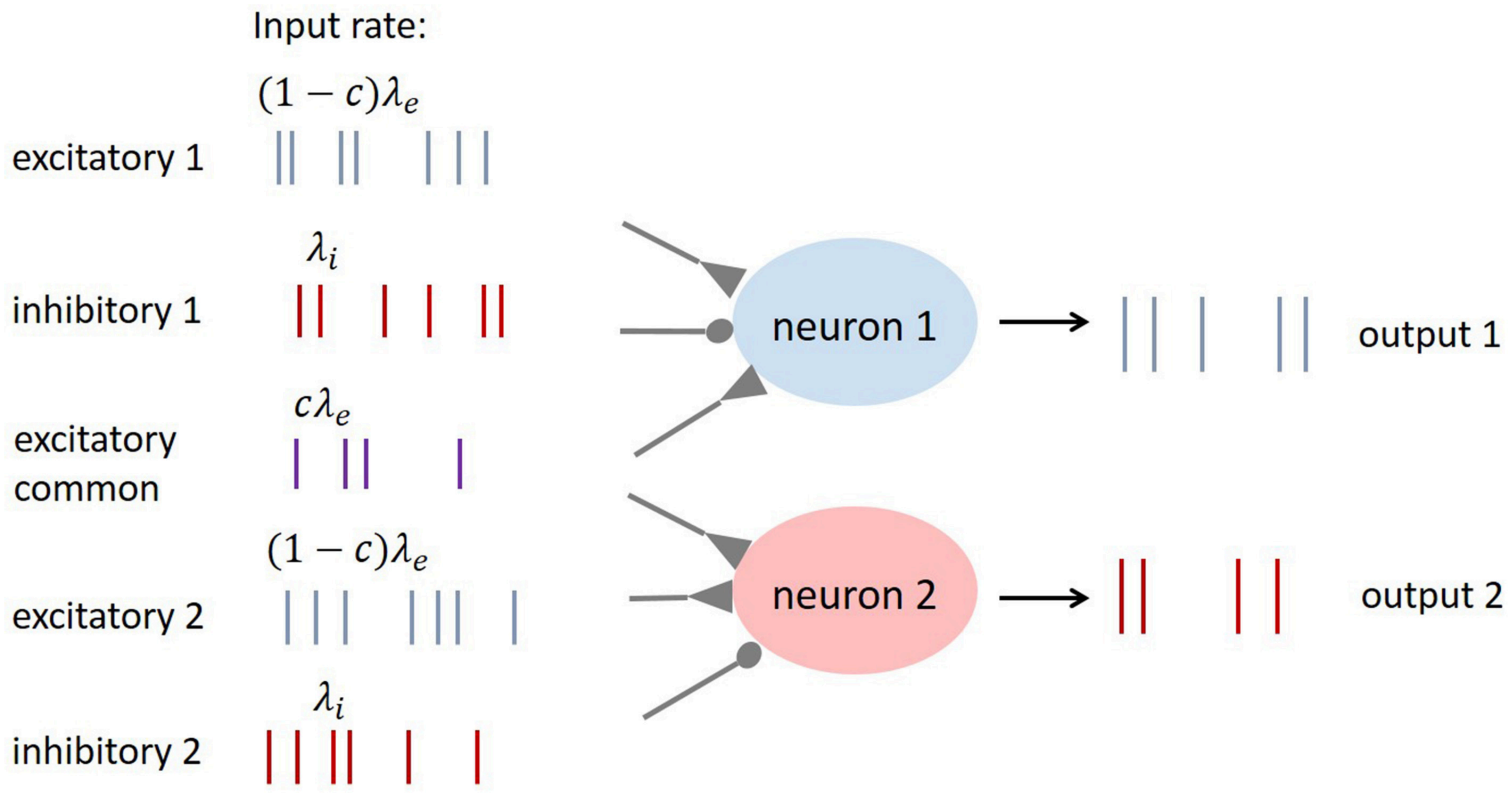
**The structure of input spike trains to the pair of neurons in study**.

In order to add correlation to the input spike trains, we adopt the Single Interaction Process (Kuhn et al., 2003). Each neuron receives an independent excitatory spike train with input rate (1 − *c*)λ_*e*_. In addition, both neurons receive a common excitatory spike train with input rate *cλ*_*e*_. The total excitatory input rate is then λ_*e*_ and the pairwise spike count correlation coefficient between the spike trains received by each neuron is *c*. Inhibitory spike trains are not correlated in this work. The input structure is illustrated in Figure 1.

### Balance between excitation and inhibition

Most neurons operate in the fluctuation driven regime, as evidenced by the highly variable inter-spike interval (ISI) in observed neural spike trains (Softky and Koch, 1993; Shadlen and Newsome, 1998). It means that excitation and inhibition are balanced. In this work, this is achieved by adjusting the inhibitory input rate λ_*i*_ such that the average output firing rate ν_*out*_, calculated from a long time window of *T* = 1.5 × 10^6^*s*, remains constant for various parameters. The reason for doing so is that output correlations are sensitive to the base-line firing rate of the post-synaptic neurons, as shown by de la Rocha et al. (2007).

### Characterizing output correlations

To quantify the correlations of the output spike trains, we consider the cross-correlation function CCF(δt), given by

(6) $$\begin{array}{rcl} \text{CCF}\left(\delta \text{t}\right) & = & \langle n_{1}\left(t\right)n_{2}\left(t+\delta \text{t}\right)\rangle -\langle n_{1}\left(t\right)\rangle \langle n_{2}\left(t+\delta \text{t}\right)\rangle \\ = & \langle n_{1}\left(t\right)n_{2}\left(t+\delta \text{t}\right)\rangle {{\;-\nu }_{out}}^{2}, \end{array}$$

where *n*_*j*_(*t*) is the number of spikes per second in a spike train of the jth neuron. The value of the cross-correlation function has the physical meaning of “the number density of extra spike pairs (as a result of correlation) per second.” Here, “extra” compares to the case where the input is totally uncorrelated, and “spike pairs” here means the concurrence of two spikes, one from each neuron, fulfiling the condition that the spike from neuron 1 precedes that of the neuron 2 by δt, where the choice of neuron 1 and neuron 2 is arbitrary but fixed once it is made.

In order to further separate quantitatively output correlations from synchrony, we introduce two quantities, *corr* and *sync*, by integrating the area below the graph of CCF from time δt = −*T*_*large*_ to *T*_*large*_ and from δt = −*T*_*small*_ to *T*_*small*_ respectively, where *T*_*large*_ (*T*_*small*_) is chosen to have a large (small) value.

(7) $$\begin{array}{rcl} corr & = & \int_{-T_{large}}^{T_{large}}\left[\langle n_{1}\left(t\right)n_{2}\left(t+\delta \text{t}\right)\rangle -{\nu_{out}}^{2}\right]\;d\left(\delta \text{t}\right)\\ = & \int_{-T_{large}}^{T_{large}}\langle n_{1}\left(t\right)n_{2}\left(t+\delta \text{t}\right)\rangle d\left(\delta \text{t}\right)-2\;T_{large}{\nu_{out}}^{2} \end{array}$$

(8) $$\begin{array}{rcl} sync & = & \int_{-T_{small}}^{T_{small}}\langle n_{1}\left(t\right)n_{2}\left(t+\delta \text{t}\right)\rangle d\left(\delta \text{t}\right)-2\;T_{small}{\nu_{out}}^{2} \end{array}$$

These quantities correspond to the number of extra spike pairs per second (as a result of correlation) in the time window *T*_*small*_ and *T*_*large*_, and hence describe the strength of output correlations and synchrony respectively.

Table 1 shows the parameters used in this work.

**Table 1:** **Parameters used in this work**.

**Parameters**	**Value**		**Unit**
**Single Neuron Dynamics**			
*V*_*r*_	−70		mV
*V*_*e*_	0		
*V*_*i*_	−75		mV
τ_*m*_	20		ms
$\frac{A_{e}}{G_{l}}$	0.1		
$\frac{A_{i}}{G_{l}}$	0.3		
τ_*i*_	8		ms
**Input statistics**			
*c*	0.2		
λ_*e*_	3000 (low), 60000 (high)		Hz
ν_*out*_	8±0.03		Hz
**Spiking mechanism (Sections Asymmetric Effects of Membrane Potential and Synaptic Integration Time Constants on Output Correlations and Slow Synaptic Filtering Induces Strong Burst Firing)**			
*V*_*th*_	−50		mV
*V*_*reset*_	−60		mV
*t*_*refract*_	2		ms
*I*_*fahp*_*max*__	0		
*I*_*sahp*_*max*__	0		
**Spiking mechanism (Section Burst Firing Greatly Enhances Output Correlations)**			
*V*_*spike*_	0		
*t*_*delay*_	0.5		ms
*V*_*th*_*rest*__	−50		ms
	λ_*e*_ = 3000	λ_*e*_ = 60000	
*V*_*th*_*max*__	−48.2	−48.8	mV
*I*_*fahp*_*max*__	−1000	−800	mA
τ_*fahp*_	1	variable	ms
*I*_*sahp*_*max*__	−40	0	mA
τ_*sahp*_	variable	N/A	
**Correlation analysis**			
*T*_*small*_	1.1		ms
*T*_*large*_	10.1		ms

### Prevalence of burst firing

To quantify the prevalence of burst firing in a spike train, we use the probability distribution of the ISIs of an output spike train, and define the prevalence of burst firing (*p*_*burst*_) of a spike train as the probability of two consecutive spikes with their ISI being less than 16 ms, given as

(9) $$\begin{array}{l} p_{burst}=\frac{N\left(t\right)|ISI<16ms}{N\left(t\right)}, \end{array}$$

where *N*(*t*) is the number of spikes in a spike train. Since the output firing rate is kept constant at 8 Hz (See Section Balance between Excitation and Inhibition), the ISI of spikes defined as a part of burst firing are smaller than one eighth of their mean. Such spikes can be considered to be significantly clustered.

### Rank correlation between burst firing and output correlation

In order to quantitatively describe how much the increase of burst firing implies that of output correlations, we calculate Spearman's rank correlation ρ (Spearman, 1904) between *p*_*burst*_ and *corr* that are obtained at various parameters, defined by

(10) $$\begin{array}{l} \rho =1-\frac{6\underset{i}{\sum }d_{i}^{2}}{n\left(n^{2}-1\right)}, \end{array}$$

where *n* is the total number of pairs of data points in the data sets (of *p*_*burst*_ and *corr*) and *d*_*i*_ is the difference in ranks between the ith data points in the two sets.

ρ describes the correlation of ranks between *p*_*burst*_ and *corr*. If ρ takes a value close to 1 (-1), it means that *corr* is “almost” monotonically increasing (decreasing) with *p*_*burst*_. Otherwise, it means that the relationship between *p*_*burst*_ and *corr* cannot be well fitted by a monotonic function.

### Numerical methods

A finite difference method is used in integration. For all numerical integration, we used Heun's method, a second-order finite difference method, with time step △*t* = 0.02 *ms*.We interpolated spike times linearly within time steps for consistency (Shelley and Tao, 2001). The model is simulated for the time $t_{total}=7.5\times 10^{6}s$, including a transient period of *t*_*tran*_ = 0.5*s* added before results are taken to allow transient effects of initial conditions to decay sufficiently. The bisection method is employed to adjust the inhibitory input rate λ_*i*_ in order to achieve constant output firing rate ν_*out*_. Numerical simulations were carried out using our own custom programs written in C++.

## Results

### Asymmetric effects of membrane potential and synaptic integration time constants on output correlations

First, we simulated pairs of conductance-based LIF neurons receiving partially common inputs under different conductance states, characterized by the level of input activities λ_*e*_, with various excitatory synaptic time constant τ_*e*_ in the absence of spike adaptation mechanisms (See Section Spiking Mechanism). Please note that we only consider cases in which neurons in the same pair have identical properties, i.e., λ_*e*_ and τ_*e*_ are the same for both neurons. The output correlations and synchrony, as quantified by *corr* and *sync* (See Methods), are obtained numerically. The results are shown in **Figure 3**. Many features of the dependence of output correlations and synchrony on τ_*e*_ and λ_*e*_ can be explained by considering the strength and the duration of the effects of an input spike on the membrane potential distribution (See Chan, 2015 for a detailed discussion and Rosenbaum and Josić, 2011 for the concept of “memory” of a spike).

How long the membrane potential is perturbed after an input spike has arrived, assuming small conductance fluctuations, mostly depends on the longer of the two time constants τ_*e*_ and τ_*eff*_. When τ_*e*_ is large and τ_*eff*_ is small, charges take time to pass through the synapses but once they do, they cause a quick rise in the membrane potential. On the other hand, when τ_*eff*_ is large and τ_*e*_ is small, charges pass through the synapses quickly, but the membrane potential takes a longer time to rise. Based on this reasoning, the effect of a single input spike on the membrane potential for neurons with small τ_*eff*_ and large τ_*e*_ should be roughly similar to their counterparts with small τ_*e*_ and large τ_*eff*_, and in a linear system this is true (Ostojic et al., 2009). If this intuition is sufficient to account for the process of correlation transfer in our model, the output correlations should be similar in these two scenarios.

**Figure 2:**
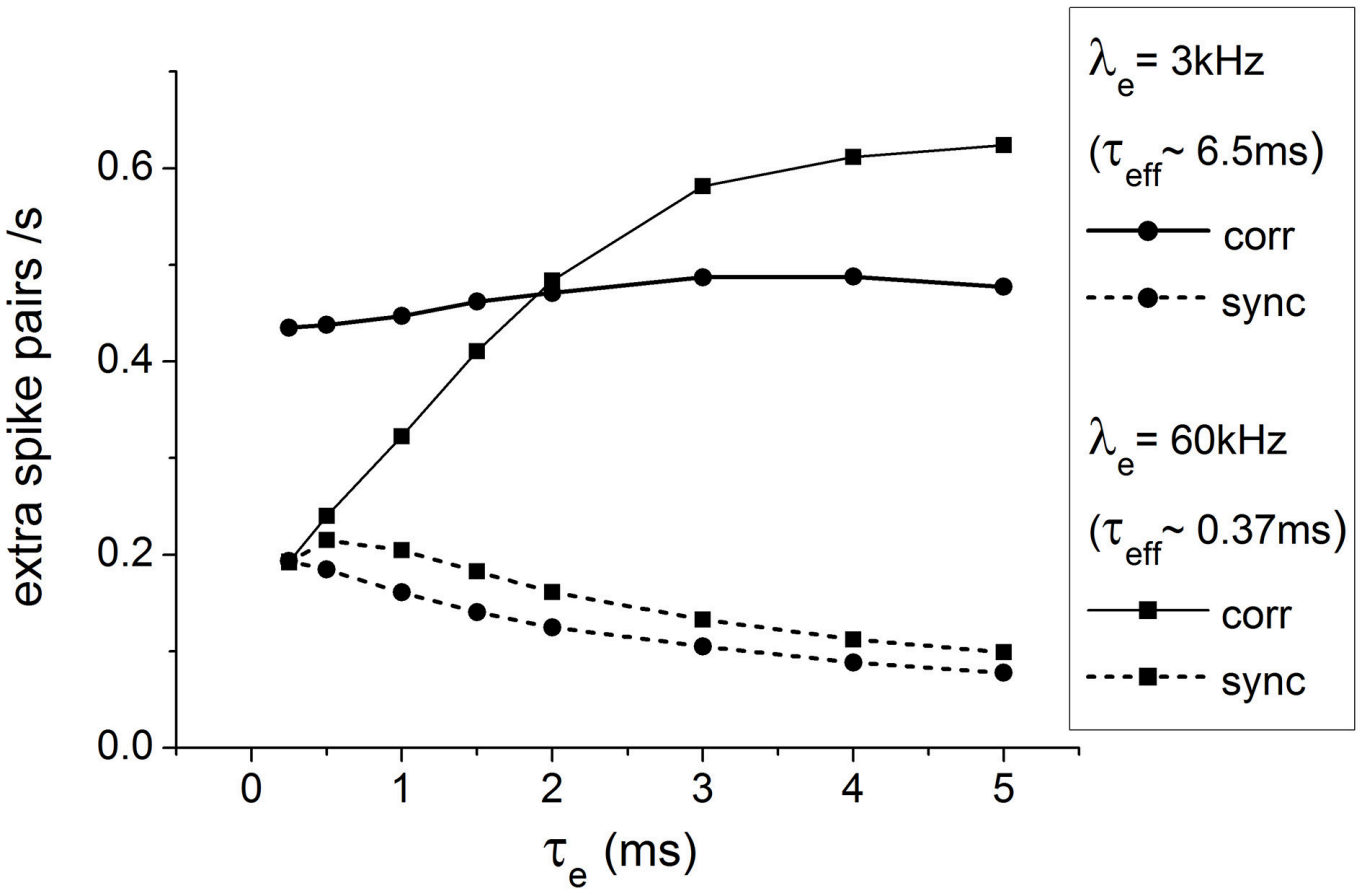
**Output correlations and synchrony of spike trains of a pair of neurons receiving correlated input**. When λ_*e*_ is high (τ_*eff*_ ≈ 0.37 *ms* and τ_*e*_ is large, the output correlation well exceeds the case when λ_*e*_ is low (τ_*eff*_ ≈ 6.5*ms*). Please refer to Section Characterizing Output Correlations for the meaning of *corr* and *sync*.

However, Figure 2 shows that when λ_*e*_ is high (τ_*eff*_ ≈ 0.37 *ms*), the output correlation increases drastically with τ_*e*_ such that at τ_*e*_ = 5 *ms*, the output correlation ends up well exceeding the output correlation in the case when λ_*e*_ is low (τ_*eff*_ ≈ 6.5 *ms*). This suggests that the effect of a single spike on the membrane potential alone cannot sufficiently account for such asymmetric effects of τ_*eff*_ and τ_*e*_ on output correlations.

### Slow synaptic filtering induces strong burst firing

**Figure 3:**
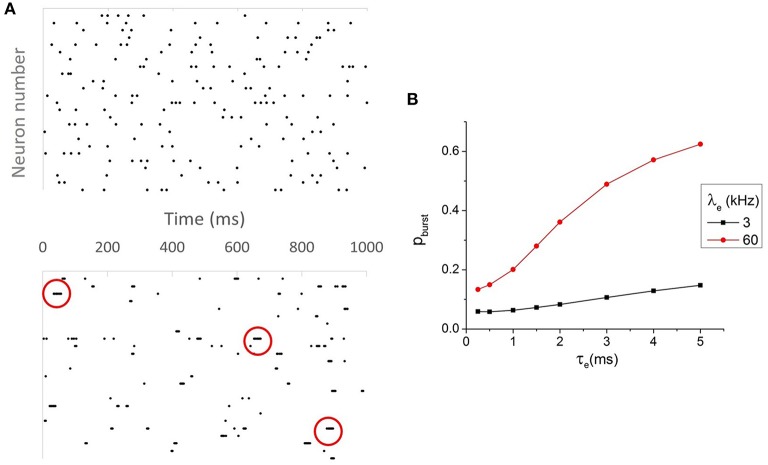
**(A)** Raster plot for neurons with τ_*e*_ = 0.5 ms and τ_*eff*_ ≈ 6.5 *ms* (top) and τ_*e*_ = 4 *ms* and τ_*eff*_ ≈ 0.37 *ms* (bottom). Neurons with small τ_*e*_ and large τ_*eff*_ almost never fire bursts, while their counterparts with large τ_*e*_ and small τ_*eff*_ burst frequently. The red circles mark some of the instances of burst firing. **(B)** The prevalence of burst firing. Neurons with large τ_*e*_ and large λ_*e*_ (or small τ_*eff*_) fire most frequently, as measured by *p*_*burst*_.

A key difference between neurons with small τ_*e*_ and large τ_*eff*_ and neurons with small τ_*eff*_ and large τ_*e*_ is that for the former, an input spike can contribute to only a single output spike, while for the latter, an input spike can contribute to multiple output spikes, since synaptic ion channels do not close when the post-synaptic neuron fires. These multiple output spikes can take place within the time scale of the synaptic time constant, which is larger than the membrane time constant but still much smaller than the average inter-spike interval of spontaneous background activities. This phenomenon is commonly known as burst firing. Figure 3A shows the raster plot of output spike trains of neurons with small τ_*e*_ and large τ_*eff*_ (top) and their counterparts with large τ_*e*_ and small τ_*eff*_ (bottom). It is clear from visual inspection that the latter frequently exhibit burst firing while the former barely show any burst. To describe the prevalence of burst firing in a spike train quantitatively, we computed the value of *p*_*burst*_ (see Methods) for pairs of neurons with different τ_*e*_ and τ_*eff*_. *p*_*burst*_ is indeed the greatest when τ_*eff*_ is small and τ_*e*_ is large, as shown in Figure 3B. Having established that burst firing distinguishes the scenarios of large and small τ_*e*_ and τ_*eff*_, we hypothesize that the burst firing may contribute to the drastic increase of output correlations in the case of large τ_*e*_, which is studied in the following section.

### Burst firing greatly enhances output correlations

**Figure 4:**
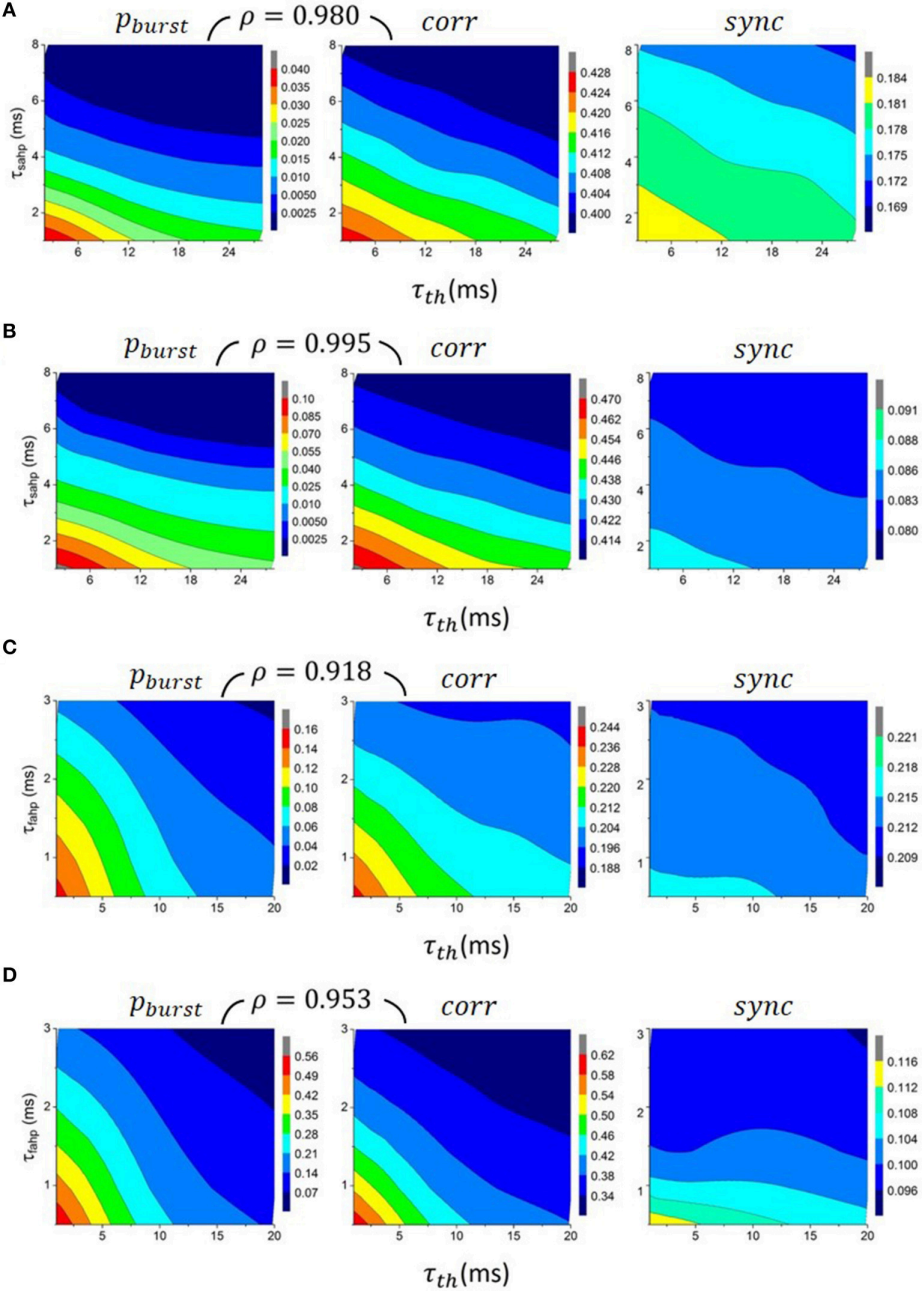
**Dependence of the prevalence of burst firing *p*_*burst*_ (left), output correlations *corr* (middle) and synchrony *sync* (right) on post-spike increase of firing threshold τ_*th*_ and on the time constants for AHP, τ_*sahp*_ (A,B) and τ_*fahp*_ (C,D)**. *p*_*burst*_ decreases with all three, τ_*sahp*_, τ_*fahp*_ and τ_*th*_, and so does *corr*, and to a lesser extent, *sync*. Spearman's rank correlation ρ between *p*_*burst*_ and *corr*, as shown above the contour plots, is very close to 1, which means that *corr* is “almost monotonically increasing” with *p*_*burst*_. **(A)** τ_*eff*_ ≈ 6.5*ms*, τ_*e*_ = 0.5 *ms*, **(B)** τ_*eff*_ ≈ 6.5 *ms*, τ_*e*_ = 4 *ms*, **(C)** τ_*eff*_ ≈ 0.37 *ms*, τ_*e*_ = 0.5 *ms* and **(D)** τ_*eff*_ ≈ 0.37 *ms*, τ_*e*_ = 4 *ms*.

Biological neurons have various adaptation mechanisms, which prevent or reduce rapid repetitive firing when a neuron receives strong excitation. After-spike hyperpolarizing currents (Storm, 1987, 1990) and increased firing threshold after post-synaptic firing (Henze and Buzsáki, 2001; Platkiewicz and Brette, 2010) are two of them. We incorporated these effects into the conductance-based LIF model in the previous section by adding hyperpolarizing input currents (AHP) and raising the firing threshold after post-synaptic firing. The currents and the raised threshold would then decay to zero or their resting values with time constants τ_*xahp*_ and τ_*th*_ respectively, as described in the Methods. It is expected that increasing τ_*xahp*_ and τ_*th*_ would make it harder for neurons to fire again within a short period after each spike, and hence reduce the prevalence of burst firing. Figures 4A–D (left) shows that the *p*_*burst*_ indeed decreases as τ_*xahp*_ and τ_*th*_ increase for neurons with different values of τ_*e*_ and τ_*eff*_. When τ_*xahp*_ and τ_*th*_ are both large, *p*_*burst*_ becomes very small, suggesting that under such conditions neurons hardly fire bursts.

If burst firing enhances output correlations, then increasing τ_*xahp*_ and τ_*th*_ should lead to a reduction of output correlations as bursts become increasingly rare. This is indeed shown to be true as illustrated in Figures 4A–D (middle). Output correlations are reduced most substantially after applying AHP currents and the variable threshold when τ_*eff*_ is small and τ_*e*_ is large, the case where the prevalence of burst firing would otherwise be the strongest. We have calculated Spearman's rank correlation coefficient ρ between *p*_*burst*_ and *corr* for neurons with the same τ_*e*_ and τ_*eff*_. It is very close to 1 regardless of τ_*e*_ and τ_*eff*_. This suggests that output correlations are “almost monotonic increasing” with burst firing.

We next asked whether the increase of output correlations due to burst firing is mostly of a long time scale (correlations) or a short time scale (synchrony)? Figures 4A–D (right) shows that while *sync* in general also decreases with τ_*xahp*_ and τ_*th*_, the scaling is much weaker than that for *corr*, both in terms of absolute value and percentage. Hence, we can conclude that burst firing strongly enhances output correlations, but only modestly enhances synchrony.

Before ending this section, we will provide some intuitive understanding on why burst firing enhances correlations. Output correlations are defined in Equation (11) as

(11) $$\begin{array}{l} corr=\int_{-T_{large}}^{T_{large}}\langle n_{1}\left(t\right)n_{2}\left(t+\delta \text{t}\right)\rangle d\left(\delta \text{t}\right)-2\;T_{large}\;{\nu_{out}}^{2} \end{array}$$

**Figure 5:**
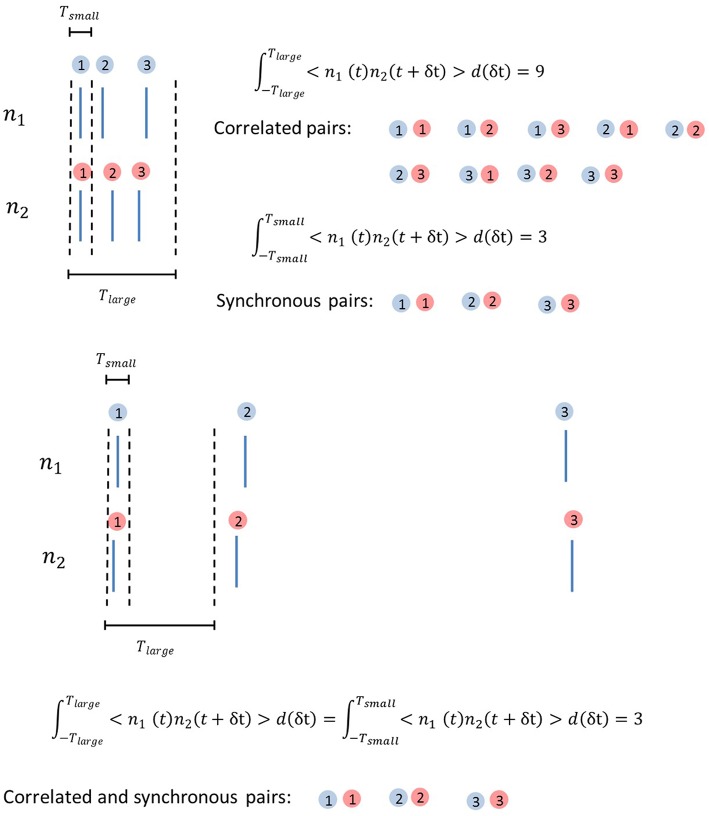
**Illustration of why burst firing enhances correlation**. Top, Correlated pairs and synchronous pairs in a burst cluster. Correlation is much enhanced due to one spike being correlated with multiple other spikes in the same burst cluster if they align well due to common inputs. Bottom, Correlated pairs and synchronous pairs with larger ISIs, having the same total number of spikes as in the top panel. The comparison of the results in the top and bottom panels shows how burst firing enhances correlation but not synchrony when neurons receive correlated input.

The second term in Equation (11) is a constant. For the first term, the non-linearity of 〈*n*_1_ (*t*) *n*_2_ (*t* + δt)〉 and large integration time window mean that if the burst clusters of the two neurons align well, a spike in a burst cluster can simultaneously be correlated to several other spikes in the same cluster. This has a multiplicative effect on correlation. Without common input, the alignment of bursts due to chance is rare. Common inputs to both neurons enhance the alignment of burst clusters, introducing strong output correlations. For synchrony, the time window is too small for this to happen. This is illustrated in Figure 5.

In this work, we only considered the case where *c* = 0.2. It has been shown in Chan (2015) that output correlation increases almost linearly with *c* in both the bursting and non-bursting cases up to *c* = 0.4. This suggests that our results should hold at least for positive value of *c* < 0.4.

## Discussion

In this work, we first showed that neurons with slow synapses exhibit much stronger output correlations than their counterparts with fast synapses, which cannot be explained by only considering the perturbation of the membrane potential by a single input spike. We found that these neurons also exhibit strong burst firing. We showed that burst firing can be reduced by spike adaptation mechanisms, namely AHP and variable spiking threshold. Using them to modulate the amount of burst firing in neurons, we were able to study how burst firing affects output correlations independently of the synaptic time scale that causes the burst firing. From the resulting analysis we conclude that burst firing greatly enhances output correlations but only moderately enhances synchrony. This offers an explanation for our initial findings of strong output correlations in neurons with slow synapses.

### Previous studies on effects of synaptic filtering and burst firing

Some previous work studied the effects of synaptic filtering on the output firing statistics analytically. Ostojic et al. (2009) considered how the time scale of synaptic filtering of a weak common input in the form of exponential excitatory post-synaptic currents (EPSCs) affects output correlations. However, in that work, the other input is assumed to be temporally uncorrelated. In such a case, the weak common input alone would not be strong enough to cause burst firing and hence the results are not comparable to our work in the regime of slow synaptic filtering. Moreno-Bote and Parga (2010) analytically derived output statistics including CCF for current-based LIF neurons with slow synapses but the role of synaptic filtering on correlation transfer remains unclear. Petrovici et al. (2013) managed to obtain expressions for the probability distribution of burst size and average inter-burst interval for neurons with slow synapses, but how such bursting influences correlation transfer remained unanswered. Our work is novel in that it provides links between burst firing and the ability of neurons to transfer correlations. These links suggest explanations why neurons with slow synapses exhibit unexpectedly strong output correlations when receiving common input, compared to their counterparts with fast synapses.

### Is enhanced correlation by burst firing due to a transient increase of output firing rate?

It has been shown that output correlation increases with firing rate (de la Rocha et al., 2007). To compensate such effects, we have ensured that the overall average output firing rate was the same in all simulations by adjusting the amount of inhibitory input (See Section Balance between Excitation and Inhibition). However, the output firing rate as measured in a small time window, i.e., the instantaneous firing rate, is fluctuating because the timing of input spikes is stochastic. If we consider bursts to be short periods in which the instantaneous firing rate is very high, it therefore seems that the observed increase of output correlations is a forgone conclusion. However, because the mean firing rate is always kept the same, there are also long periods of relative quiescence between bursts when the instantaneous firing rate is very low compared to the non-bursting case. The momentary increase of correlation due to increased instantaneous firing rate during bursts might well have been offset by the long periods of reduced rate and hence reduced correlations during the quiescent periods between bursts. Our results show that this is not the case.

In other words, our results can be interpreted in the following way: given a constant mean firing rate, increasing the variance of the instantaneous firing rate transfers input correlations to higher output correlations.

### Effects of burst firing on downstream neurons

We have shown that with correlated inputs, neurons who fire in bursts show stronger output correlations than those who do not, given they have the same synaptic and membrane time constants. In a biological neural network, the spike trains of neurons firing bursts may become input to downstream neurons. Such spike trains are non-Poisson with a high probability of having small ISIs (Ostojic, 2011). How then do these bursting, non-Poisson spike trains affect the correlation transfer in the downstream neurons? For neurons with both fast synaptic filter and short membrane integration time window, their excitatory postsynaptic potentials (EPSPs) have a shorter time scale than the ISIs within a burst, which is bounded from below by the absolute refractoriness. Therefore, spikes within a burst are processed no different from other spikes and the correlation transfer should be little different from the case where the input obeys Poisson statistics.

**Figure 6:**
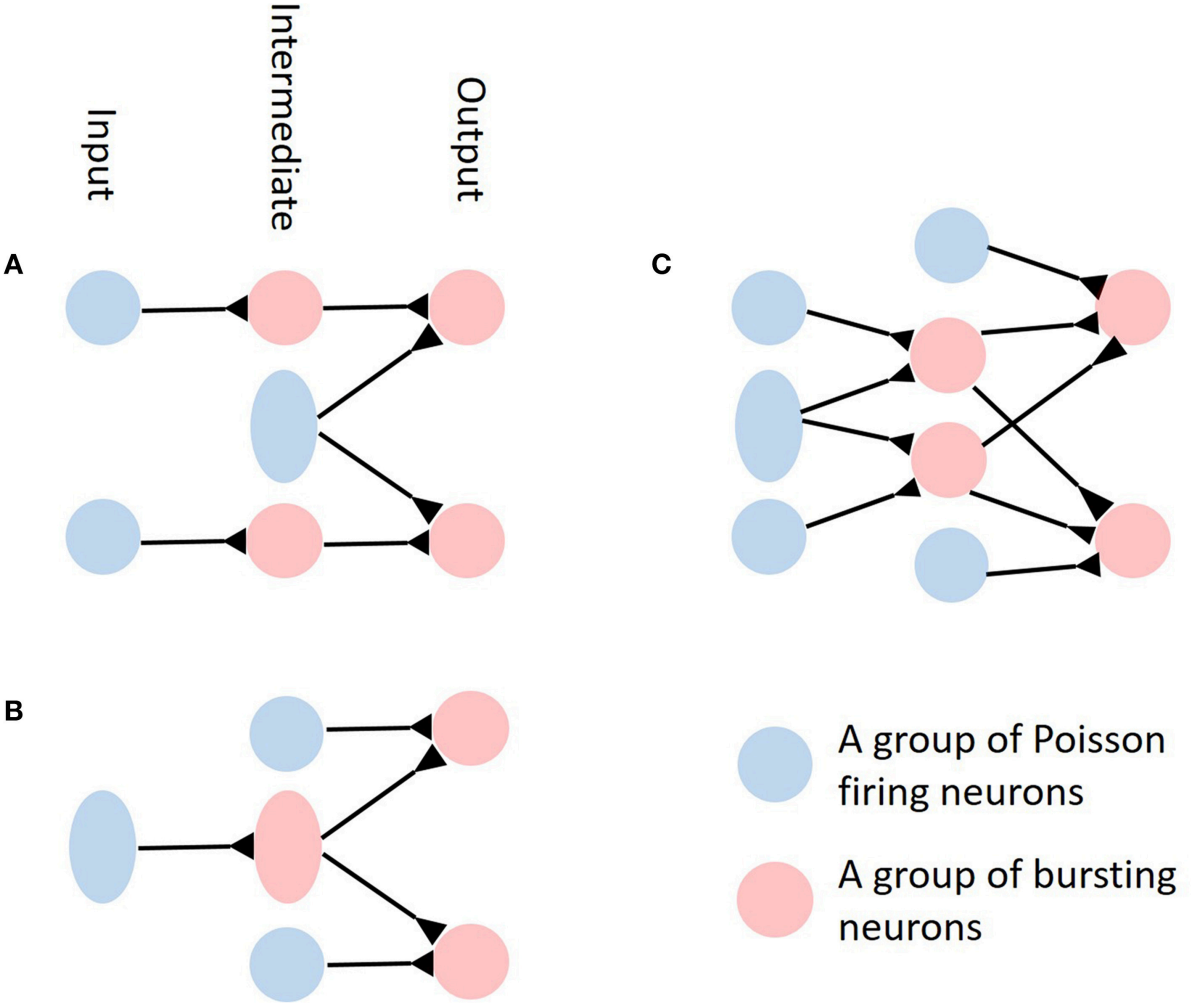
**Simplified illustrations of possible neural networks with correlated or uncorrelated bursting neurons and Poisson inputs**. **(A)** Bursting spike trains are independent to downstream neurons while the downstream neurons receive additional, correlated Poisson input. In the diagram, blue circles represent groups of Poisson firing neurons while red circles represent groups of bursting neurons. Inhibitory connections are not shown in the diagram. **(B)** Neural network where the bursting spike trains generated by neurons receiving independent input constitutes the correlated input to downstream neurons. **(C)** Same as **(B)** but the bursting spike trains now receive correlated input and therefore are correlated themselves.

If the time scale of either the synaptic filtering or membrane integration is slow, then there are at least three interesting scenarios to consider. In the first scenario, the bursting input to downstream neurons is independent and the downstream neurons receive additional, correlated Poisson input, as depicted in Figure 6A. We hypothesized that the downstream neurons would exhibit burst firing but such burst firing would be uncoordinated among neurons so would not have significant effects on output correlations. In the second scenario, the bursting spike trains are generated by neurons receiving independent inputs but they project to several downstream neurons, so becoming correlated inputs to the downstream neurons, as depicted in Figure 6B. In this case, the bursts would form a strong transient excitatory drive simultaneously to more than one downstream neuron, much like the correlated synchronous inputs in Schultze-Kraft et al. (2013). Therefore, we expect that like in that work, the output correlations and synchrony would be boosted. The last scenario would be the same as the previous scenario except that the bursting spike trains are generated by neurons already receiving correlated inputs, as depicted in Figure 6C. In this case, not only the bursts would drive the downstream neurons in coordination, but the resulting drive would also be much stronger than in the previous case since they arrive in synchrony. From the results of Schultze-Kraft et al. (2013) we hypothesize that the output correlation would be further strengthened compared to the previous case because of the increase of synchrony in the input. The role of burst firing on correlation transfer in more complex biological neural networks of multiple layers is an interesting direction for future work.

### Burst firing and correlation transfer in biologically realistic neural networks with inhibition

In this work, we showed on the single neuron level that slow synaptic filtering gives rise to burst firing while spike adaptation mechanisms suppress it. In biological neural networks, interactions between excitation and inhibition may also contribute to the enhancement or suppression of burst firing. For instance, in networks with feedforward inhibition where input from a layer of neurons simultaneously excites the neurons in the output layer as well as inhibitory interneurons which are also connected to the above-mentioned neurons, output neurons can only fire within a few milliseconds after receiving strong excitatory input, as the strong excitation activates the interneurons which then silenced the output neurons (Pouille and Scanziani, 2001; Kremkow et al., 2010). This could result in the suppression of otherwise burst firing in the absence of inhibition. Indeed, it has been suggested that feedforward inhibition may be responsible for the transition between the burst firing state and the suppressed state in the subiculum (Sah and Sikdar, 2013).

Previous work has shown that feedforward inhibition (Middleton et al., 2012), as well as recurrent inhibition (Tetzlaff et al., 2012) and lateral inhibition (Giridhar et al., 2011), play important roles in modulating the level of correlation in neural networks by suppressing and reshaping the output correlations of neurons receiving correlated inputs. In light of it would be interesting to study whether and how burst firing is involved in the changes of output correlations observed in such inhibitory circuits.

### Implication on neural coding

Neurons with slow synapses are integrators (König et al., 1996; Hong et al., 2012; Ratté et al., 2013). Previous work (Hong et al., 2012; Ratté et al., 2013) has shown that correlations of integrators arises from co-modulation of rate and is of long time scale, which is in agreement with the results the work presented here (See Figure 3). These previous studies further claimed that such correlations do not provide temporal information about the input and reduce coding efficiency through introducing redundancy. This is not so evident in our case where the correlations are mostly attributable to burst firing. Several experimental studies (Snider et al., 1998; Lesica and Stanley, 2004; Oswald et al., 2004; Eyherabide, 2008) and numerical studies (Kepecs and Lisman, 2003; Lesica and Stanley, 2004; Oswald et al., 2004) have shown that information may be represented in neural bursts. Transmission of information by bursts is advantageous since bursts can reliably produce output spikes (Snider et al., 1998), thereby reducing information loss when output neurons fail to produce spikes due to noise and synaptic failure (Krahe and Gabbiani, 2004). Moreover, input bursts can better produce precisely timed output than isolated spike (Kepecs and Lisman, 2003) such that correlated bursting spike trains may constitute precisely timed, synchronous input to downstream neurons. Therefore, the large correlation-to-synchrony ratio observed in neurons with slow synapses does not necessarily imply that such neurons cannot employ temporal based coding. It is possible that the presence of bursts actually facilitates information transmission through storing information in the bursts themselves or increasing the efficiency of other codes.

## Author contributions

HC: Design the work, run the simulation, do the data analysis, produce the figures, write the manuscript. DY: Design the work, write the manuscript. CZ: Design the work, write the manuscript. TN: Write the manuscript.

### Conflict of interest statement

The authors declare that the research was conducted in the absence of any commercial or financial relationships that could be construed as a potential conflict of interest.
